# Comparison of Outcomes of Percutaneous Coronary Intervention Versus Coronary Artery Bypass Grafting

**DOI:** 10.7759/cureus.12202

**Published:** 2020-12-21

**Authors:** Ratan Kumar, Kheraj Mal, Muhammad Khalid Razaq, Mansoor Magsi, Muhammad Khizar Memon, Sidra Memon, Sana Irfan, Kanwal Bansari, Basma Ali, Amber Rizwan

**Affiliations:** 1 Cardiology, Khairpur Medical College, Nawabshah, PAK; 2 Cardiology, National Institute of Cardiovascular Diseases, Sukkur, PAK; 3 Cardiology, Sheikh Zayed Medical College, Rahim Yar Khan, PAK; 4 Internal Medicine, Taluka Hospital Kandhkot, Kandhkot, Kashmore, PAK; 5 Internal Medicine, Liaquat University of Medical and Health Sciences, Hyderabad, PAK; 6 Internal Medicine, Jinnah Sindh Medical University, Karachi, PAK; 7 Family Medicine, Jinnah Post Graduate Medical Center, Karachi, PAK

**Keywords:** percutaneous coronary intervention, coronary artery bypass grafting, myocardial infarction

## Abstract

Introduction

Percutaneous coronary intervention (PCI) and coronary artery bypass grafting (CABG) are two common treatment options used in patients suffering from coronary artery disease. Selection and favorability of one over the other depend on individual clinical scenarios. The purpose of this study is to compare outcomes after treatment with PCI and CABG.

Methods

This longitudinal observational study was conducted from April 2018 to July 2019 in a cardiovascular unit of a tertiary care hospital. Participants who were eligible for revascularization were randomized either to receive stent (PCI) or surgery (CABG). Patients were then followed up for 12 months for the development of all-cause mortality and major adverse cardiovascular events (MACEs).

Results

At 12 months, patients randomized to the PCI group had an increased risk of repeat revascularization (21.3% vs. 7.4%; p = 0.007), whereas a similar number of patients in both groups died (3.8% vs. 3.7%), suffered myocardial infarction (7.6% vs. 5.6%), or had a cerebrovascular accident (3.8% vs. 2.8%).

Conclusions

This study showed that PCI had an increased risk of repeat revascularization compared to CABG. However, both had comparable significance in the development of MACEs. Nevertheless, there is a need for further study to better assess the outcomes of either, especially in the long run.

## Introduction

Currently, both percutaneous coronary intervention (PCI) and coronary artery bypass grafting (CABG) are reliable therapeutic options for patients with left main coronary disease. Treatment selection should consider the extent of the disease (favoring CABG for severe lesions) and patient preference (usually favoring PCI) [1]. The clinical efficacy of PCI has been established in acute ischemia and in other limited situations. However, accumulating evidence shows that patients with complex atherosclerotic lesions, multivessel disease, left main stem disease, left ventricular dysfunction (LVD), and diabetes mellitus (DM) derive more benefit from CABG than from PCI. Therefore, CABG should be presented to these patients as the more effective intervention in terms of survival, freedom from re-intervention, and cost-effectiveness [2]. However, patients prefer not to undergo surgery because of a fear of complications and of physical and mental debilitation because of surgery [3].

In patients deemed inoperable for CABG with a high-risk profile, a suboptimal outcome was achieved after PCI. In patients who are not candidates for PCI, bypass surgery produces excellent results [4]. Kappetein et al. found after three-year follow-up that patients with more complex disease have an increased risk of an all-cause mortality and major adverse cardiovascular events (MACEs) with PCI, and CABG is the preferred treatment option [5]. In left main coronary artery disease (CAD), CABG reduced major adverse cardiac or cerebrovascular events at five years compared with PCI with drug-eluting stents [6]. In a randomized controlled trial (RCT), after 10 years, CABG in older patients was associated with fewer subsequent coronary interventions, whereas PCI treatment was associated with a higher incidence of myocardial infarction (MI) [7].

The need to assess outcomes of PCI and CABG is vital in appropriate treatment selection according to each individual’s relevant conditions and to better understand which modality will result in better outcomes that may help to improve the quality of life and mortality of these patients in the future.

## Materials and methods

This longitudinal observation study was conducted from April 2018 to July 2019 in a cardiovascular unit of a tertiary care hospital. A total of 220 participants were randomized by 1:1 ratio using an online software Research Randomizer (https://www.randomizer.org) to either receive stent through PCI (n = 103) or to undergo CABG (n = 107). The procedures were explained to each participant. Inclusion criteria included patients with silent, stable, or unstable angina, and the presence of at least two lesions in different coronary arteries, eligible for revascularization. All patients or their attendants gave written, informed consent.

Patients were followed up for 12 months or till the development of all-cause mortality and MACEs, whichever came first. In this study, MACEs were defined as death, cerebrovascular accident, MI, and revascularization. Overall, 10 patients were lost to follow-up; seven from the PCI group and three from the CABG group. Patients who completed the study were included in the final analysis.

Statistical analysis was performed using SPSS version 23.0 (IBM Corp., Armonk, NY, USA). Continuous variables were presented as mean and SD. Binary outcomes were expressed as frequencies and percentages and were compared in terms of relative risk with 95% CI.

## Results

Both groups, PCI and CABG, had a similar demographic and risk factor profile (Table 1).

**Table 1:** Comparison of demographic data and risk factor profile between PCI and CABG CABG, coronary artery bypass grafting; MI, myocardial infarction; PCI, percutaneous coronary intervention.

Characteristics	PCI (n = 103)	CABG (n = 107)
Age (years)	59 ± 9	59 ± 10
Male, %	62	61
Diabetes, %	20	21
Hypertension, %	44	43
Hypercholesterolemia, %	61	62
Current smoker, %	25	25
Previous MI, %	31	32
Unstable angina, %	32	31

At 12 months, patients randomized to the PCI group had an increased risk of repeat revascularization (21.3% vs. 7.4%, p = 0.007), whereas a similar number of patients in both groups died, or suffered MI or cerebrovascular accident (Table 2).

**Table 2:** Comparison of outcomes between PCI and CABG CABG, coronary artery bypass grafting; NS, nonsignificant; PCI, percutaneous coronary intervention.

Event	PCI (n = 103)	CABG (n = 107)	Relative risk (95% CI)	p-Value
Death	4 (3.8%)	4 (3.7%)	1.03 (0.26-4.04)	NS
Cerebrovascular accident	4 (3.8%)	3 (2.8%)	1.38 (0.31-6.03)	NS
Myocardial infarction	8 (7.6%)	6 (5.6%)	1.35 (0.48-3.78)	NS
Repeat revascularization	22 (21.3%)	8 (7.4%)	2.85 (1.33-6.12)	0.007

## Discussion

PCI and CABG are considered revascularization procedures, but only CABG has been proven to provide long-term prognostic benefits in patients with CAD [2,8]. Myocardial infarcts are usually generated by non-flow-limiting stenosis. PCI is mainly focused on treating flow-limiting stenosis, and thus it cannot limit the formation of new infarcts. CABG, on the other hand, can significantly limit the formation of new infarcts by providing flow distal to the vessel occlusion [8].

This study was conducted to assess the outcomes of PCI and CABG in patients with silent, stable, or unstable angina, and the presence of at least two lesions in different coronary arteries eligible for revascularization. Patients in both groups (PCI and CABG) had similar demographic and risk factor profile including age (59 ± 9 vs. 59 ± 10), male gender (62% vs. 61%), DM (20% vs. 21%), hypertension (44% vs. 43%), hypercholesterolemia (61% vs. 62%), present smoking history (25% vs. 25%), history of MI (31% vs. 32%), and unstable angina (32% vs 31%). After 12 months of follow-up, patients in the PCI group had an increased risk of repeat revascularization (22.1% vs. 7.4%, p = 0.007) as compared to patients in the CABG group. However, MACEs including mortality rate and incidence of both MI and cerebrovascular accident were similar in both groups in our study. Similar findings were reported by Palmerini et al., who concluded that both PCI and CABG had similar long-term composite rates of mortality, MI, and cerebrovascular disease, with PCI demonstrating early 30-day safety advantage and CABG procedure showing lesser rates of unplanned revascularization [9].

Rosenfeldt et al. in their study analyzed recent evidence to conclude that CABG is more beneficial than PCI in patients with complex atherosclerotic lesions, multivessel disease, left middle circumflex artery (LMCA) disease, LVD, and DM. However, patients still usually prefer PCI because the procedure is minimally invasive [2]. In a systemic review that included 13 RCTs and five meta-analyses to demonstrate the effectiveness of PCI and CABG, Deb et al. concluded that CABG should be recommended in patients with DM, LMCA disease, multivessel CAD, or LVD, and SYNTAX score >22 (severe CAD) due to improved long-term survival and lower cardiac events, whereas PCI is considered in patients with less complex CAD (SYNTAX ≤ 22) or if the patient is at a high surgical risk [10]. Patients with multivessel involvement or LMCA reported greater relief from angina after six and 12 months of undergoing CABG compared to PCI [11].

The study has its limitations. It was a single-center study and hence sample size diversity was reduced. Patients were not followed up for a longer duration, as most of them came from the peripheral area and there was a strong chance that they might be lost to follow-up. As our study was limited to a 12-month follow-up, there is room to investigate outcomes over a longer period of time to further learn about the consequences of PCI and CABG in the long run. To the best of our knowledge, this is the first local study comparing the outcome of PCI vs. CABG. It will add to the limited local data we have related to cardiovascular outcomes associated with two techniques and assist clinicians in making an informed decision.

## Conclusions

It can be concluded that PCI could lead to an increased risk of revascularization compared to CABG. However, there is no superiority of one over another in terms of having a risk of MI, cerebrovascular accident, or death after PCI or CABG. These findings suggest CABG to be slightly more favorable, but it highly depends on the patient’s consent, requirement, and severity of the disease.
